# The Syk-Coupled C-Type Lectin Receptors Dectin-2 and Dectin-3 Are Involved in *Paracoccidioides brasiliensis* Recognition by Human Plasmacytoid Dendritic Cells

**DOI:** 10.3389/fimmu.2018.00464

**Published:** 2018-03-20

**Authors:** Nycolas Willian Preite, Claudia Feriotti, Dhêmerson Souza de Lima, Bruno Borges da Silva, Antônio Condino-Neto, Alessandra Pontillo, Vera Lúcia Garcia Calich, Flávio Vieira Loures

**Affiliations:** ^1^Departamento de Imunologia, Instituto de Ciências Biomédicas, Universidade de São Paulo, São Paulo, Brazil

**Keywords:** paracoccidioidomycosis, plasmacytoid dendritic cells, C-type lectin receptors, dectin-2, dectin-3, type I IFNs, inflammasome

## Abstract

Plasmacytoid dendritic cells (pDCs), which have been extensively studied in the context of the immune response to viruses, have recently been implicated in host defense mechanisms against fungal infections. Nevertheless, the involvement of human pDCs during paracoccidioidomycosis (PCM), a fungal infection endemic to Latin America, has been scarcely studied. However, pDCs were found in the cutaneous lesions of PCM patients, and in pulmonary model of murine PCM these cells were shown to control disease severity. These findings led us to investigate the role of human pDCs in the innate phase of PCM. Moreover, considering our previous data on the engagement of diverse Toll-like receptors and C-type lectin receptors receptors in *Paracoccidioides brasiliensis* recognition, we decided to characterize the innate immune receptors involved in the interaction between human pDCs and yeast cells. Purified pDCs were obtained from peripheral blood mononuclear cells from healthy donors and they were stimulated with *P. brasiliensis* with or without blocking antibodies to innate immune receptors. Here we demonstrated that *P. brasiliensis* stimulation activates human pDCs that inhibit fungal growth and secrete pro-inflammatory cytokines and type I IFNs. Surprisingly, *P. brasiliensis-*stimulated pDCs produce mature IL-1β and activate caspase 1, possibly *via* inflammasome activation, which is a phenomenon not yet described during pDC engagement by microorganisms. Importantly, we also demonstrate that dectin-2 and dectin-3 are expressed on pDCs and appear to be involved (*via* Syk signaling) in the pDC-*P. brasiliensis* interaction. Moreover, *P. brasiliensis*-stimulated pDCs exhibited an efficient antigen presentation and were able to effectively activate CD4^+^ and CD8^+^ T cells. In conclusion, our study demonstrated for the first time that human pDCs are involved in *P. brasiliensis* recognition and may play an important role in the innate and adaptive immunity against this fungal pathogen.

## Introduction

Paracoccidioidomycosis (PCM), the most prevalent and endemic deep mycosis in Latin America, is caused by the thermally dimorphic fungus *Paracoccidioides brasiliensis*. The resistance to this disease was linked to the secretion of Th1 cytokines in humans and murine models of PCM, while reduced Th1 immunity and the predominant secretion of Th2 cytokines associate with systemic and progressive illness (1, 2). The role of Th17 immunity is poorly defined. Nevertheless, in PCM patients, IL-17-expressing cells have been observed in cutaneous and mucosal lesions and they have been associated with the development of granulomas (3). Moreover, the varied patterns of T cell responses in patients with PCM lead to diverse clinical manifestations. In infected asymptomatic individuals, the resistance to infection was shown to be mediated by the Th1 response, which is essential for macrophage activation and fungal killing, while the juvenile form, the most severe form of the disease, presents a dominant Th2/Th9 response and an elevated antibody response. In adult form of the disease, a chronic inflammatory response involving Th1 and Th17-mediated immunity was also described (2).

Plasmacytoid dendritic cells (pDCs) are a DC subset largely involved in immune responses against viruses (4, 5). Following Toll-like receptors (TLRs) stimulation, the pDCs mature and become potent antigen-presenting cells (APCs), upregulating MHC and costimulatory molecules, and secreting high levels of cytokines such as IFN-α and TNF-α. Activated mature pDCs prime naive T cells for Th1 or Th17 differentiation (6, 7). Outside of viral infections, important roles for pDCs have been described in the immune response against fungal infections (8–12). A pioneer study showed that human pDCs interact to and inhibit the growth of *Aspergillus fumigatus*-hyphae. Moreover, *in vivo* depletion of pDC confers hyper-susceptibility to aspergillosis in mice. In addition, it was also demonstrated that dectin-2, a C-type lectin receptor (CLR) expressed by human pDCs, acts in collaboration with the FcRγ chain to recognize hyphae of *A. fumigatus*. This interaction induces the synthesis of TNF-α and IFN-α, and facilitates efficient antifungal activity. Additionally, pDCs stimulated by *A. fumigatus* hyphae exhibit a characteristic gene expression signature, leading to the formation of extracellular traps (pETs) (10). *P. brasiliensis* was also shown to be recognized by murine pDCs. *In vitro* stimulation of bone marrow-derived DCs (BMDCs) from susceptible (B10.A) mice induces a prevalent inflammatory myeloid phenotype characterized by the secretion of high levels of IL-12, TNF-α, and IL-1β. In contrast, in BMDCs from the resistant (A/J) mice a varied population of myeloid cells and pDCs secreting inflammatory cytokines and expressing high levels of secreted and membrane-bound TGF-β were observed following BMDCs stimulation with fungal cells (8). Importantly, in 24 of 46 PCM patients, the presence of pDCs was verified when their cutaneous lesions were immunostained with anti-pDC specific antibodies (11). In addition, peripheral blood pDCs appeared in lower numbers in PCM patients compared to the number of blood pDCs found in health donors, suggesting that pDCs migrate to the lymph nodes, spleen, and target organs during a *P. brasiliensis* infection (12). Taking in account these findings and the fact that human innate immunity against *P. brasiliensis* infection is poorly defined, we aimed to investigate the interaction of human pDCs with*P. brasiliensi*s yeast cells. Moreover, considering our previous data on the engagement of TLRs, complement receptor-3, and CLRs in *P. brasiliensis* recognition (13–17), we considered that it would be important to define the class of innate receptors involved in fungal recognition by human pDCs. Here, we show that when stimulated by *P. brasiliensis* yeast cells human pDCs are activated, produce inflammatory cytokines, and acquire enhanced fungicidal activity. In addition, we demonstrate that *P. brasiliensis* recognition by human pDCs is mediated by dectin-2 and dectin-3, and is regulated by Syk signaling. Our study of the secretion of mature IL-β and caspase-1 activation has also indicated that *P. brasiliensis* recognition triggers inflammasome activation in human pDC, a finding that, as far we know, has not been described previously. Finally, our data also suggested that *P. brasiliensis* activated pDCs can prime the activation of CD4^+^ and CD8^+^ T cells, and may participate in the acquired immunity to this pathogen.

## Materials and Methods

### Ethics Statement

All research involving human participants was approved by the Institute of Biomedical Science Institutional Ethics Committee. Written informed consent was obtained from all human participants and all clinical investigations were conducted according to the principles expressed in the Declaration of Helsinki.

### Isolation of Human pDCs, mDCs, and CD3^+^ Cells

The human pDCs were isolated from healthy donors using magnetic beads as previously described (10). About 120 mL of the peripheral blood was collected by venipuncture. The blood samples was anticoagulated with heparin, and the peripheral blood mononuclear cells (PBMCs) were purified by Ficoll-Hypaque density gradient centrifugation. The PBMCs were stained with CD304-coated magnetic beads (Miltenyi Biotec) and the pDCs were isolated after two rounds of positive selection. For flow cytometric experiments in which pDCs were gated using an anti-CD123 antibody, only one round of positive selection was run. For some experiments, the flow-through cells (here called pDC^−^ cells), which consisted of PBMCs depleted of CD304^+^ cells, were also collected. For coculture experiments, purified human myeloid dendritic cells (mDCs) and CD3 cells were obtained from PBMCs by positive selection using magnetic beads (Miltenyi Biotec). mDCs were also used in qPCR experiments. The purity of the cell populations was confirmed by assessing the expression of CD123, CD1c, and CD3 as pDC, mDC, and lymphocyte markers, respectively, by flow cytometry. The purity of pDCs, mDCs, and CD3^+^ cells always exceeded 95%.

### *P. brasiliensis* Yeast Cells and pDC Stimulation

*Paracoccidioides brasiliensis* yeast cells were maintained by weekly cultivation in Fava Netto culture medium at 37°C and used on day 7 of culture. The highly virulent *P. brasiliensis* 18 isolate was used in this study. To determine the viability of fungal cell suspensions we used Janus Green B vital dye (Merck), and the viability was always greater than 95%. For colony-forming units (CFU) and ELISA experiments, pDC (1 × 10^5^) or flow through-cells (pDC^−^) were challenged overnight with *P. brasiliensis* in different ratios of pDC:*P. brasiliensis*, such as 10:1, 25:1, and 50:1 (1 × 10^4^, 4 × 10^3^, and 2 × 10^3^ yeasts, respectively) as indicated in the figure legends.

### Reagents and Cell Culture

The RPMI-1640 media was obtained from GIBCO (Invitrogen) and was supplemented with 100 U/mL penicillin, 100 U/mL streptomycin, and 2 mM l-glutamine. IgG Rat monoclonal anti-dectin-1 (Biolegend), dectin-2 (R&D System), dectin-3 (Biolegend), and antimincle (InvivoGen) blocking antibodies were used in the concentration indicated in the figure legends. The TLR-9 antagonist (A151, TTAGGG oligonucleotide sequence, 10 µg/mL), TLR-7/9 inhibitor Bafilomycin A1 (10 µg/mL), and the immunostimulatory CpG 2336 oligonucleotide (100 ng/mL)were purchased from InvivoGen. Ultrapure *Escherichia coli* LPS (10 ng/mL) and the Syk inhibitor piceatannol (10 µM) was purchased from Sigma.

### *P. brasiliensis*–pDC Interaction

*Paracoccidioides brasiliensis* yeast cells were labeled with fluorescein isothiocyanate (FITC) as previously described (18). Briefly, the yeasts were washed in phosphate-buffered saline (PBS, pH = 7.4) and heat-killed at 60°C for 1 h. To eliminate the aggregates, the yeast cells suspension was subjected to three cycles of sonication for 10 s each (21% amplitude) with Sonics (Vibra Cell VCX 750, Sonics & Materials). The yeasts were washed twice with PBS, counted and adjusted to 1 × 10^6^ cells/mL, and then incubated with FITC (100 µg/mL, Sigma) for 25–30 min at 36°C. The yeast suspension was then washed with PBS twice. The pDCs (2 × 10^5^) were challenged for 4 h with *P. brasiliensis-*FITC (1 × 10^5^) at a ratio of 2:1 at 36°C in 5% CO_2_ to permit fungi adhesion and ingestion. The cells were then harvested and the pDCs were labeled with anti-CD123 (eBioscience) antibody for 25 min at 4°C. Because *P. brasiliensis* yeast cells are highly variable in size and granularity (different sizes, number of buds, and number of nuclei), the granulocyte gates defined by size (FSC) and granularity (SSC) in order to determine the pDC population were not used. A minimum of 50,000 events were acquired on a FACScanto II flow cytometer (BD Biosciences) using the FACSDiva software (BD Biosciences). The data were analyzed using the FlowJo software (Tree Star).

### Cytokine Measurements

The pDCs were cultured in 96-well plates in pDC media. The cells were left untreated or incubated with anti-dectin-1, dectin-2, dectin-3, or antimincle antibodies (100 µg/mL) for 30 min at 37°C. The pDCs were then challenged with *P. brasiliensis* yeast cells at a final volume of 200 µL in pDC media at the ratios indicated in the figure legends. Control wells contained pDCs only, pDCs and antibodies, pDCs and CpG, or pDCs and LPS. For the inhibition of TLR-9, pDCs were left untreated or incubated for 30 min with either 10 µg/mL of bafilomycin A1 or 10 µg/mL of the TLR-9 antagonist A151. After 18 h of incubation at 37°C, the supernatants were removed and the levels of TNF-α, IL-6, IL-1β, IFN-α, and IFN-β were measured by ELISA according to the manufacturers’ protocols (eBioscience for TNF-α and IL-6; Biolegend for IL-1β; PBL Assay Science for IFN-α and IFN-β).

### CFU Assays

The number of viable microorganisms in cell cultures was performed by counting the number of CFU as previously described (19). Briefly, after 18 h of coculture, the plates were centrifuged (400 *g*, 10 min, 4°C), and the supernatant was collected for cytokine measurements by ELISA. The wells were lysed with 200 µL of distilled water. The suspensions were collected in individual tubes and centrifuged (400 *g*, 10 min, 4°C). The pellet was suspended in 1 mL of PBS, and 100 µL of each lysate was spread onto BHI medium. The colonies (CFU) were counted for 15 days.

### pDC Flow Cytometric Analysis

The pDC^+^ populations (2 × 10^5^/well) were either left unstimulated or challenged overnight with *P. brasiliensis* yeast cells (4 × 10^3^). For cell-surface staining, the pDCs were washed and suspended at 2 × 10^5^ cells/mL in staining buffer (PBS, 2% fetal calf serum, and 0.1% NaN_3_) and then stained in the dark for 20 min at 4°C with the optimal dilution of anti-123, CD86, MHC-II antibodies. The cells were then washed twice with staining buffer, suspended in 200 µL of paraformaldehyde (PFA) to fix the cells. To measure caspase-1 activity, the pDCs were adjusted to 2 × 10^5^ viable cells in 20 µL of apoptosis wash buffer. Cells were stained with FLICA probe according to the manufacturer’s protocol (Immunochemistry Technologies) for 1 h at 37°C in 5% CO_2_. Control wells contained untreated pDCs, pDC and CpG, or pDC and LPS. In some experiments, cells were treated with 1 mM ATP (Sigma-Aldrich) for 15 min and then stained with FLICA probe. Active caspase-1 was then measured by flow cytometry as previously described (16). To exclude dead cells, the Live/Dead Fixable Violet Cell Stain Kit (Life Technologies) was used according to the manufacturer’s instructions. A minimum of 50,000 events were acquired on a FACScanto II flow cytometer (BD Biosciences) using the FACSDiva software (BD Biosciences). The pDCs were gated based upon their forward and side light scatter. The cell surface expression of pDC markers was analyzed using FlowJo software (Tree Star).

### RNA Isolation and Relative Gene Expression Analysis

Total RNA was isolated and converted into cDNA from 2 × 10^5^ purified pDC^+^ and mDC^+^, unstimulated or challenged overnight with *P. brasiliensis* yeast cells (4 × 10^3^), using the TaqMan Gene Expression Cells-to-CT kit according to manufacturer’s protocol (Applied Biosystems, Thermofisher Scientific). The expression of *CLEC7A/DECTIN1, CLEC6A/DECTIN2, CLEC4D/DECTIN3, CLEC4E/MINCLE* was evaluated using gene-specific TaqMan assays (Applied Biosystems) and qPCR on a MxP3000P Real-Time PCR System (Stratagene). Raw expression data (Ct) were normalized with the expression of the housekeeping gene glyceraldehyde-3-phosphate dehydrogenase/*GAPDH* (ΔCt). Relative expression of the target genes was calculated as 2^−ΔCt^ according to Schmittgen and Livak (20), comparing stimulated and unstimulated cells (ΔΔCt = ΔCt*_Pb_* − ΔCt_Unstimulated_).

### pDC/mDC and Lymphocyte Coculture

The pDCs^+^ or mDCs^+^ populations (1 × 10^5^/well) were challenged with *P. brasiliensis* yeast cells (1 × 10^4^) for 2 h and cocultured with CD3^+^ cells (1 × 10^6^) for 5 days at 37°C in 5% CO_2_. All cells populations were isolated from the same donor. The ratio DCs to lymphocytes (1:10) was determined in a previous study (15). For cell-surface staining, the cocultured cells were washed and suspended at a concentration of 5 × 10^5^ cells/mL in staining buffer (PBS, 2% fetal calf serum and 0.1% NaN_3_). They were then stained in the dark for 20 min at 4°C with the optimal dilution of anti-CD4, CD8, CD25, and anti-CD69 antibodies. The cells were then washed twice with staining buffer, and suspended in 200 µL of PFA to fix the cells. A minimum of 50,000 events were acquired on FACScanto II flow cytometer (BD Biosciences) using the FACSDiva software (BD Biosciences). The lymphocytes were gated based upon forward and side light scatter. The cell surface expression of lymphocyte markers was analyzed using FlowJo software (Tree Star).

### Statistical Analysis

For comparisons of two groups, the means ± SE were analyzed using the two-tailed unpaired Student’s *t*-test with the Bonferroni correction applied when making multiple comparisons. For comparisons of more than two groups, significance was determined using the one- or two-way analysis of variance with the Tukey multiple corrections. Kruskal–Wallis test was applied in RT-PCR experiments. All data were collected and analyzed from at least three independent experiments. The levels of significance were *p* < 0.05 (*p < 0.05, **p < 0.01, and ***p < 0.001). Calculations were performed using a statistical software package (GraphPad Prism 6.0).

## Results

### The *P. brasiliensis*–pDC Interaction and Fungicidal Activity

**Figure 1:**
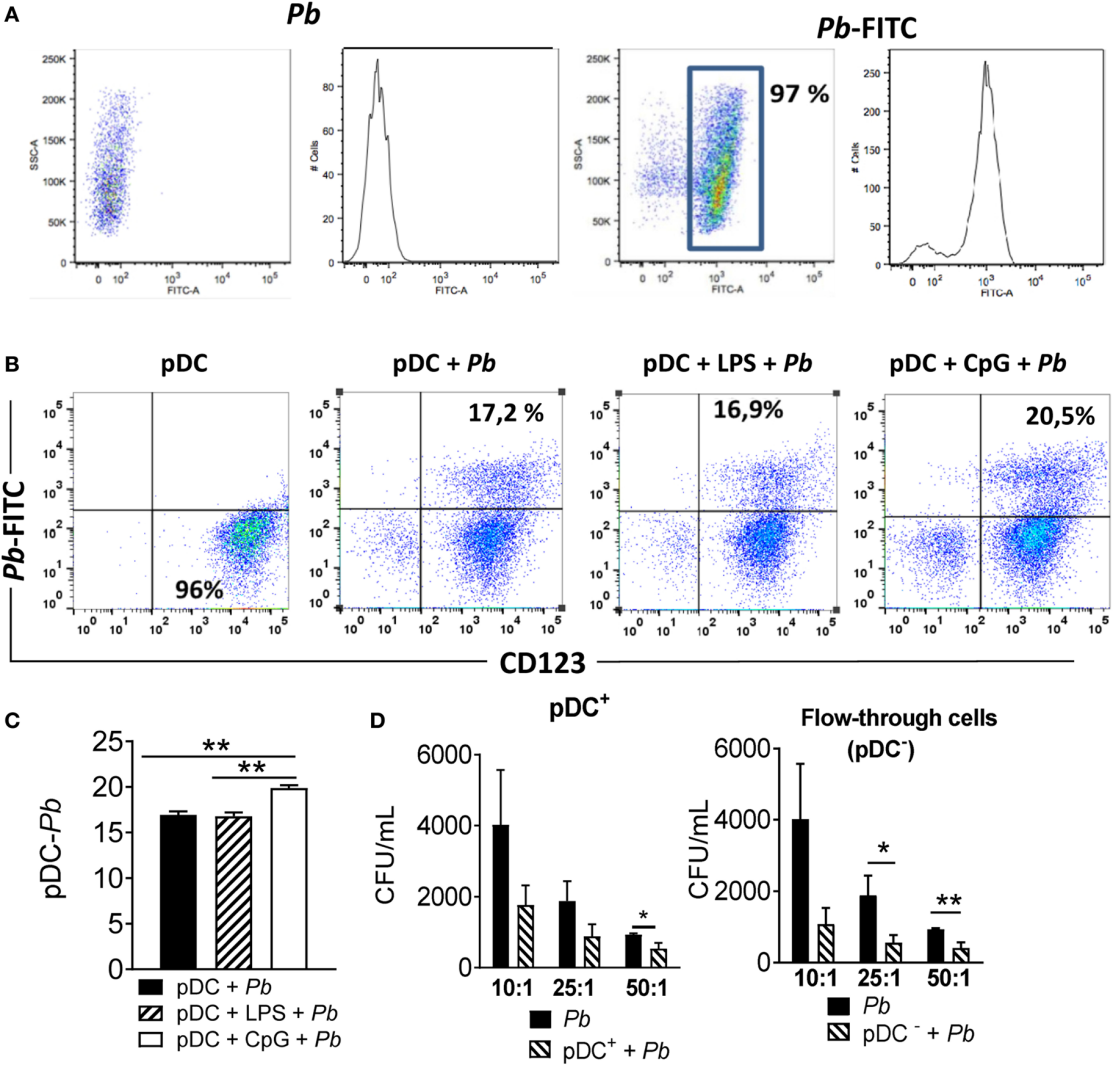
Plasmacytoid dendritic cells (pDCs) interact with and control *Paracoccidioides brasiliensis* growth. **(A,B)** Peripheral blood mononuclear cells (PBMCs) were separated into pDC positive (pDCs+) using magnetic beads conjugated to an anti-CD304 antibody. The cells (2 × 10^5^/well) were challenged for 4 h with *P. brasiliensis* yeast cells (2 × 10^5^/well) previously labeled with FITC. Some wells were treated with CpG (100 ng/mL) or LPS (10 ng/mL) before the challenge. After 4 h of culture, the cells were labeled with an anti-CD123 antibody (specific to human pDCs) in the appropriate titration, and then 50,000 events were acquired by flow cytometry. **(B)** Dot plots are representative results for an experiment from three independents experiments. **(C)** Data represent means ± SE of the pDC–*P. brasiliensis* interaction from three donors, tested in triplicate **p < 0.01. **(D)** For CFU analysis, PBMCs were separated into pDC-positive (pDCs^+^) and pDC-negative (pDC^−^) fractions using CD304-coated magnetic beads. The pDCs^+^ and flow through-cells (1 × 10^5^/well) were then challenged with *P. brasiliensis* yeast cells in different ratios of pDC: *Pb*, such as 10:1, 25:1 and 50:1 (1 × 10^4^, 4 × 10^3^, and 2 × 10^3^ yeasts, respectively). After 18 h of culture, the plates were centrifuged and the supernatant was collected for cytokine measurements by ELISA. The pellet was lysed and suspended in 200 µL of phosphate-buffered saline. Next, 100 µL was transferred to BHI medium and the colonies (CFU) counted for 15 days. Data represent means ± SE of CFU from four donors, tested in triplicate. *p < 0.05, **p < 0.01, and ***p < 0.001.

Initially, we investigated the ability of human pDCs to interact and phagocytose *P. brasiliensis* yeast cells. PBMC-isolated pDCs were challenged with FITC-conjugated *P. brasiliensis* (Figure 1A) for 4 h, and the frequency of adhered or ingested *P. brasiliensi*s yeast cells by pDCs was determined by counting the number of labeled pDC (CD123^+^ cells) containing *Pb*-FITC (CD123^+^ *Pb*-FITC^+^). Figure 1B shows that about 17% of the total pDCs were double-positive for CD123 and *Pb*-FITC, suggesting that pDCs interacted with *P. brasiliensi*s yeast cells. Considering that human pDCs are TLR-9^+^ and TLR-4^−^ (21, 22), the TLR-4 bacterial LPS agonist and the TLR-9 CpG agonist were used in some experiments to specifically prime pDCs to enhance their phagocytic ability. As expected, the ability of pDCs to interact with the yeasts did not significantly change in the presence of LPS. However, the addition of CpG enhanced the *P. brasilienis*–pDC interaction (Figures 1B,C).

To analyze the fungicidal ability of human pDCs, the PBMCs were separated into pDC^+^ or pDC^−^ populations. The pDC^−^ fraction is composed of the flow-through cells resulting from the pDC isolation. This fraction, used as a control in some assays, contains mostly non-pDC PBMCs such as conventional DCs, monocytes, lymphocytes and small fractions of other cell subpopulations. The depletion of pDC was able to reduce the frequency of pDC on the PBMCs in about 90% (Figure S1 in Supplementary Material) resulting in less than 0.2% of pDC in the PBMCs. The cells were challenged overnight with *P. brasiliensis* yeast cells in different pDC: *Pb* ratios (10:1, 25:1, and 50:1). Figure 1D shows that only in the 50:1 ratio were pDCs able to control fungal growth. The pDC^−^ fractions, however, showed fungicidal activity at ratios of 25:1 and 50:1.

### Cytokine Release by Human pDCs Stimulated by *P. brasiliensis* Yeast Cells

**Figure 2:**
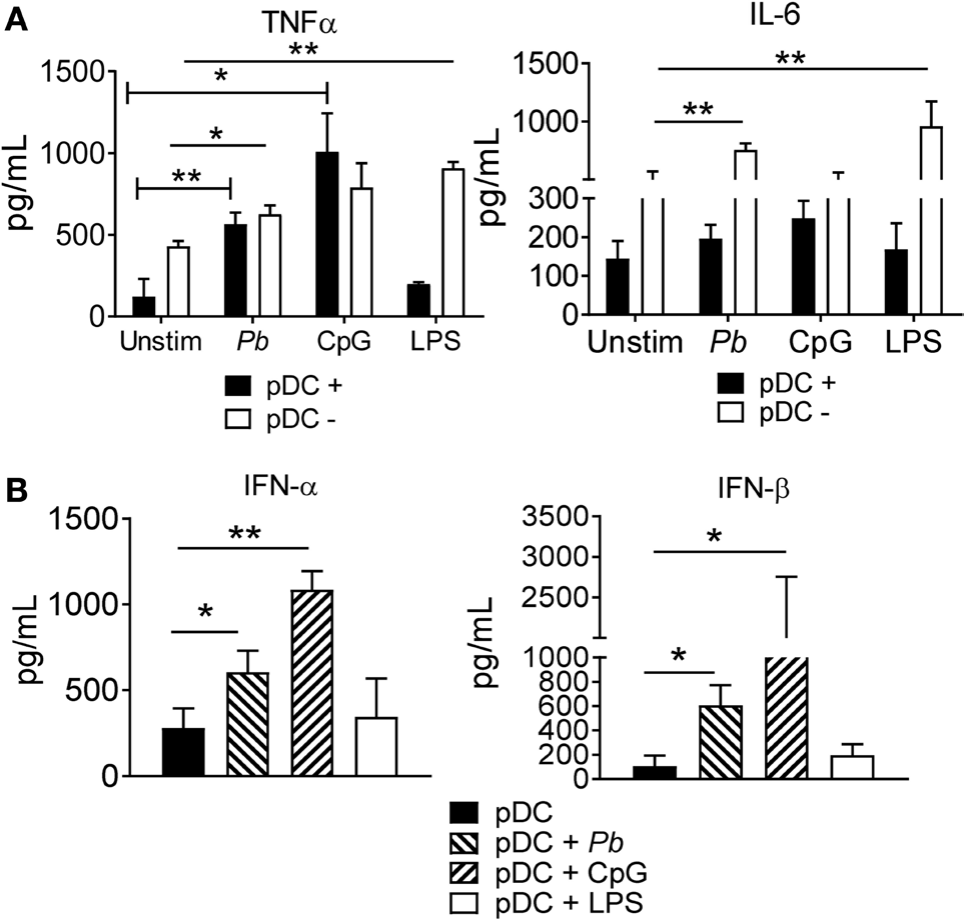
Proinflammatory and type I IFNs cytokines released by plasmacytoid dendritic cells (pDCs) stimulated with *Paracoccidioides brasiliensis* yeast cells. Peripheral blood mononuclear cells (PBMCs) were separated into pDC-positive (pDCs^+^) and pDC-negative (pDCs^−^) populations using magnetic beads conjugated to anti-CD304 antibodies. The cells (1 × 10^5^/well) were left without stimulation or challenged overnight with of *P. brasiliensis* yeast cells (2 × 10^3^), CpG (100 ng/mL), or LPS (10 ng/mL). The supernatant was removed and the levels of IL-6, TNF-α **(A)**, and type I IFNs **(B)** were analyzed by ELISA. Data are represented as the mean ± SE of the average cytokine concentrations from five donors, tested in duplicate. ***p < 0.001 by comparing the data indicated by the bar.

Next, we examined whether the interaction between the pDCs and *P. brasiliensis* yeast cells could induce the secretion of inflammatory cytokines. The pDCs were challenged with *P. brasiliensis* for 18 h, and the levels of TNF-α and IL-6 in the culture supernatants were determined. Both cytokines were secreted by pDCs, but only TNF-α levels were increased by *P. brasiliensis-*stimulated pDCs. The TLR-9 ligand, CpG, strongly stimulated the pDCs, whereas the TLR-4 ligand, LPS, failed to stimulate these cells to produce either TNF-α or IL-6. As expected, LPS stimulated the pDC^−^ fraction (which contained LPS-responsive monocytes) to release TNF-α and IL-6 (Figure 2A).

Next, we examined whether the interaction of pDCs with *P. brasiliensis* yeast cells could increase type I IFN release. Both IFN-α and IFN-β were secreted by pDCs^+^ following stimulation with *P. brasiliensis* yeast cells. Once again, CpG strongly stimulated the pDCs, while LPS failed to activate these cells (Figure 2B).

### *P. brasiliensis* Induces Production of IL-1β and Increases Caspase-1 Activity in Human pDCs

An emerging area of investigation examines the participation of the inflammasome in fungal infections (16, 23). The inflammasome is a multiprotein cytoplasmic complex assembled after an innate immune receptor [e.g., NOD-like receptor family, pyrin domain-containing (NLRP) 1, NLRP3, or absent in melanoma 2 (AIM2)] is activated by a pathogen-associated molecular pattern (PAMPs) or damage-associated molecular pattern. This results in a multi-protein platform that activates caspase-1, which processes pro-IL-1β and pro-IL-18 into their biologically active or mature forms (24).

**Figure 3:**
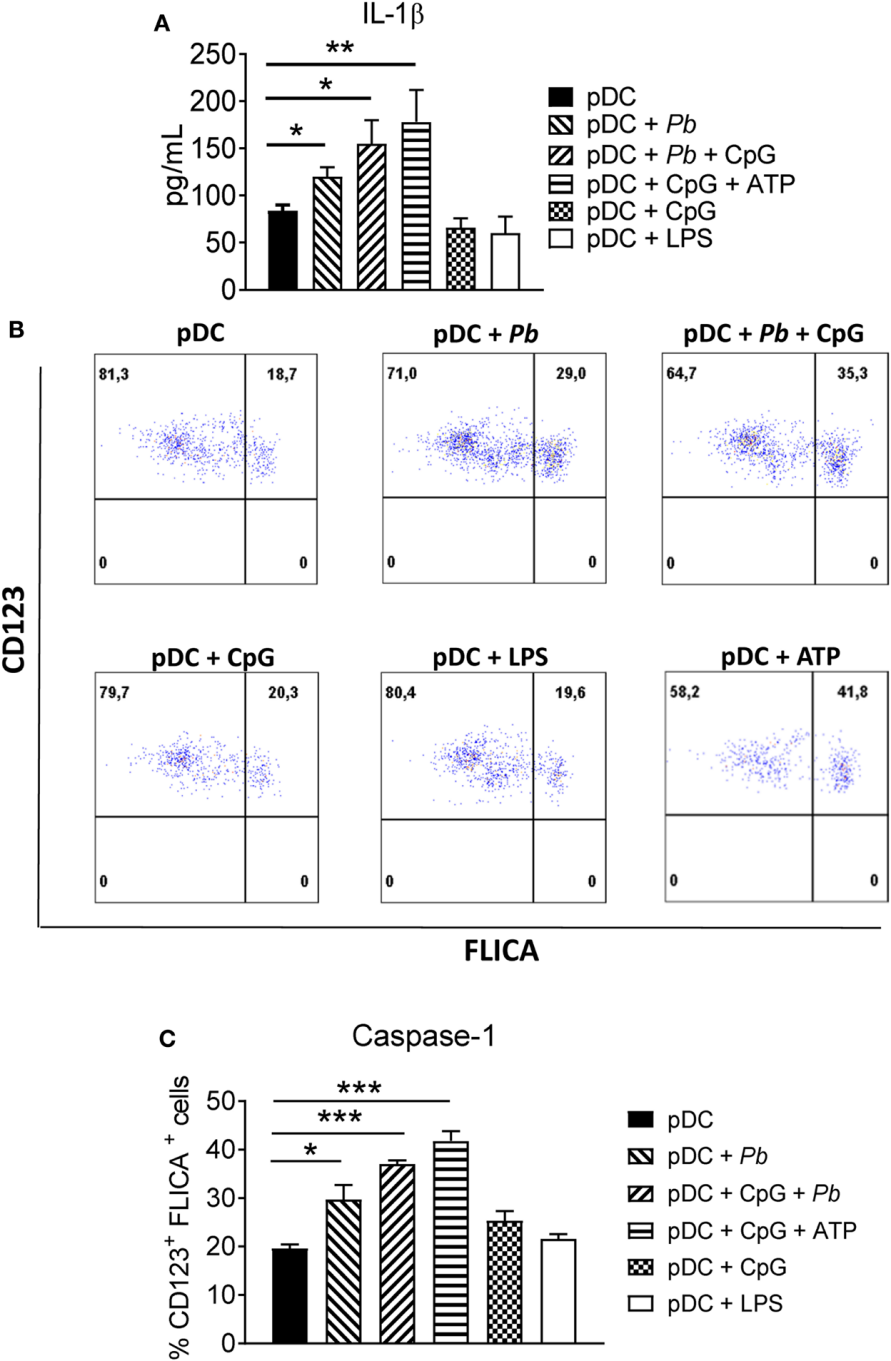
Plasmacytoid dendritic cell (pDC) exposed to *Paracoccidioides brasiliensis* yeast cells secretes mature IL-1β and express activated caspase-1. **(A)** Peripheral blood mononuclear cells (PBMCs) were separated into pDC positive (pDCs+) cells using magnetic beads conjugated to anti-CD304 antibodies. The cells (1 × 10^5^/well) were then left without stimulation or challenged overnight with *P. brasiliensis* yeast cells (2 × 10^3^), CpG (100 ng/mL), or LPS (10 ng/mL). The supernatant was removed and the levels of IL-1β were analyzed by ELISA. Data are represented as the mean ± SE of the average cytokine concentrations from three donors, tested in duplicate. *p < 0.05 by comparing the data indicated by the bar. **(B,C)** Caspase-1 activity was evaluated by flow cytometry. PBMCs were separated into pDC positive (pDCs^+^) populations using magnetic beads conjugated to anti-CD304 antibodies. The cells (2 × 10^5^/well) were left without stimulation or challenged overnight with *P. brasiliensis* yeast cells (4 × 10^3^), CpG (100 ng/mL), or LPS (10 ng/mL). Cells were stained with FLICA probe for 1 h and the percentage of CD123^+^FLICA^+^ cells were determined by flow cytometry. **(B)** Dot plots are representative results of an experiment from two independent experiments. **(C)** Data are represented as the mean ± SE of CD123^+^FLICA^+^ cells from two donors, tested in duplicate. *p < 0.05, **p < 0.01, ***p < 0.001 by comparing the data indicated by the bars.

In our experiments, *P. brasiliensis* yeast cells induced a significant production of IL-1β by pDCs. Importantly, treatment with CpG or LPS alone did not significantly increase IL-1β production by these cells (Figure 3A). Accordingly, caspase-1 activation was strongly induced in *P. brasiliensis*-stimulated pDCs, more than in LPS-treated or CpG-treated pDCs (Figures 3B,C). However, when pDCs received both signals (CpG and yeasts), enhancement in the activity of caspase-1 was observed. Importantly, the addition of 1 mM ATP to the cultures increased caspase-1 activity and IL-1β production, suggesting that a purinergic signaling could be involved in inflammasome activation by *P. brasiliensis*. To our knowledge, increased extracellular ATP activates NLRP3-inflammasome through the binding on P2 × 7 purinergic receptor inducing a K^+^ efflux which in turn activates NLRP3 (25). Deeper investigations are needed to confirm the involvement of ATP-P2 × 7-NLRP3 axis in pDC response to *P. brasiliensi*s, although the involvement of P2 × 7 receptor in the immunoprotection of murine PCM was demonstrated in our previous studies (26).

### The Role of TLR-9 in the *P. brasiliensis*–pDC Interaction

**Figure 4:**
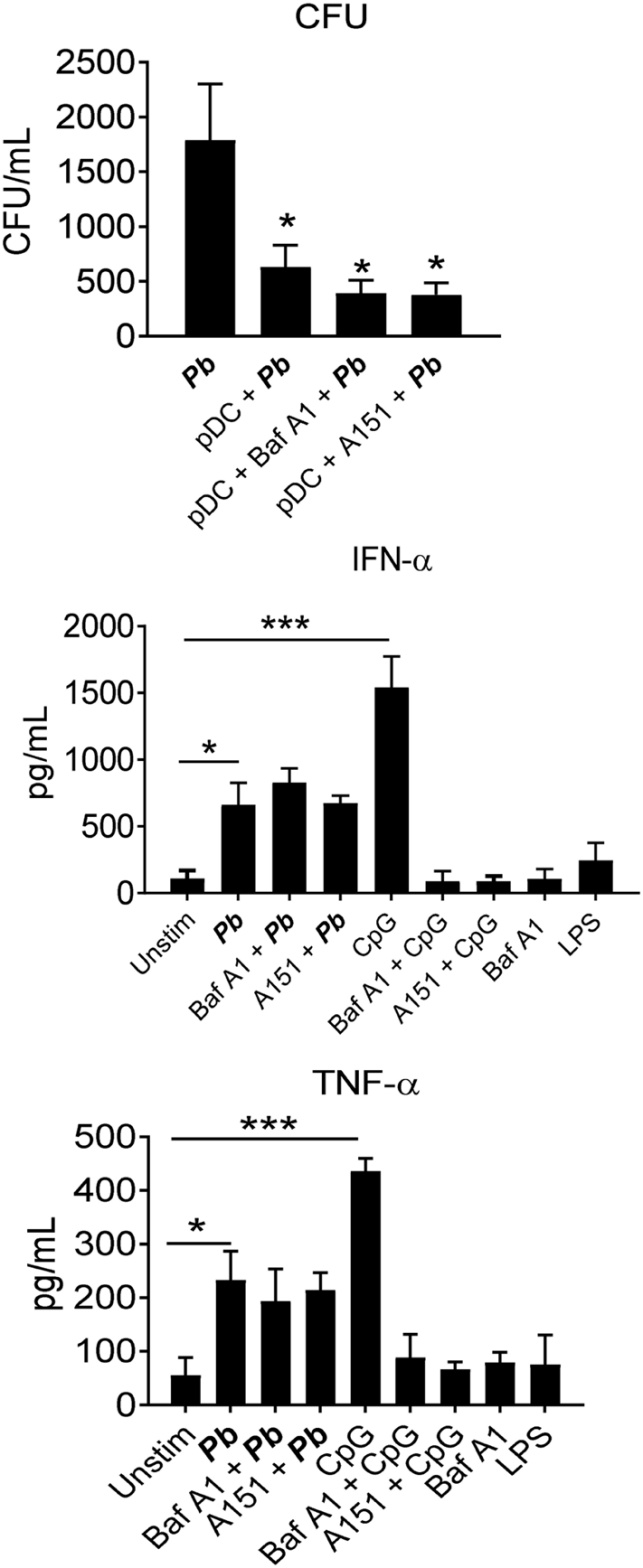
Toll-like receptor (TLR)-9 is not used by plasmacytoid dendritic cells (pDCs) to sense *Paracoccidioides brasiliensis* yeast cells. peripheral blood mononuclear cells were separated into pDC-positive populations (pDCs^+^) using magnetic beads conjugated to anti-CD304 antibody. The cells (1 × 10^5^/well) were challenged overnight with *P. brasiliensis* yeast cells (2 × 10^3^). Some wells were kept unstimulated, with only the yeasts, and LPS (100 ng/mL). Some cultures were treated with an TLR-9 antagonist (A151, TTAGGG, 10 µg/mL) or with the TLR-9 inhibitor Bafilomycin A1 (10 µg/mL) before challenging either with the yeasts or with the positive control for TLR-9 activation CpG (100 ng/mL). After 18 h of culture, the plates were centrifuged and the supernatant was collected for cytokine measurements by ELISA. The pellet was lysed and suspended in 200 µL of phosphate-buffered saline. Next, 100 µL was transferred to BHI medium, and the colonies (CFU) were counted for 15 days. Data represent the means ± SE of the CFU and cytokine concentrations from three donors, tested in triplicate. *p < 0.05 comparing the group maintained only with the fungus with the others.

Several studies have demonstrated that TLR-9 is an important PRR used by pDCs during their interaction with microorganisms. We have assessed the role of TLR-9 in the sensing of *P. brasiliensis* by human pDCs using two reagents that inhibit this receptor. Thus, the influence of bafilomycin A1, an endosomal acidification inhibitor known to interfere with TLR-9 signaling (27), and the TLR-9 antagonist A151 (28), on the ability of pDCs to kill *P. brasiliensis* and release cytokines was determined. Both TLR-9 inhibitors showed no effects on *P. brasiliensis* growth or IFN-α secretion. Furthermore, bafilomycin A1 did not affect TNF-α production. As expected, both inhibitors abrogated CpG-stimulated type I IFN release (Figure 4). These data suggest that *P. brasiliensis* is recognized by human pDCs in a TLR-9-independent manner.

### The Role of CLRs in the *P. brasiliensis*–pDC Interaction

**Figure 5:**
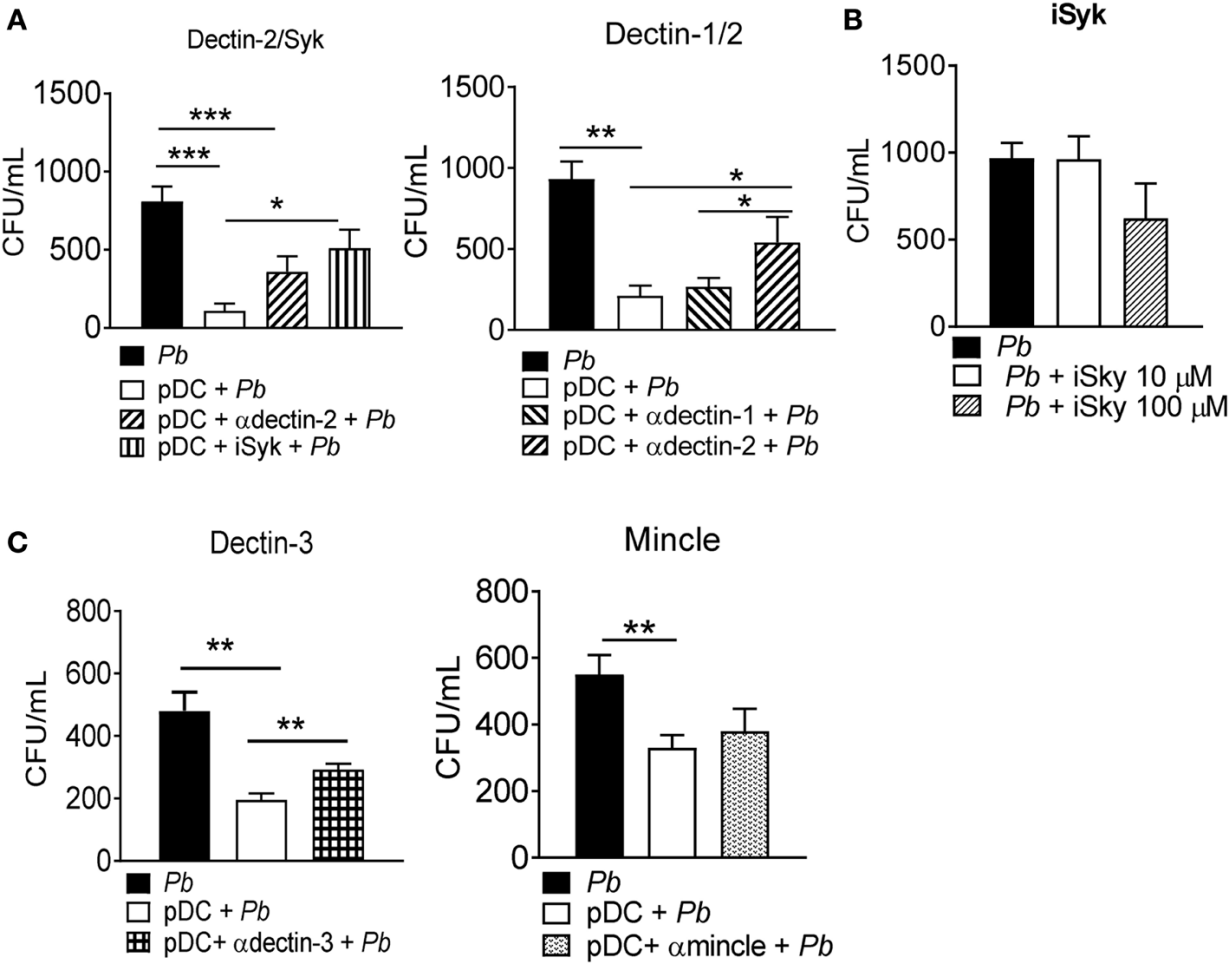
Dectin2, dectin-3, and Syk signaling activate the fungicidal activity of plasmacytoid dendritic cells (pDCs). Peripheral blood mononuclear cells were separated into pDC-positive populations (pDCs+) using magnetic beads conjugated to anti-CD304 antibody. The cells (1 × 10^5^/well) were challenged overnight with *P. brasiliensis* yeast cells (2 × 10^3^). **(A)** Some cultures were treated with anti-dectin-1, dectin-2, or with a Syk pathway inhibitor, piceatannol (10 µM). **(B)** The Syk inhibitor (10 or 100 µM) was also cultured with *P. brasiliensis* yeast cells only. **(C)** Futher, anti-mincle and anti-dectin-3 antibodies (100 µg/mL) were also used, before challenging with *P. brasiliensis* yeast cells. After 18 h of culture, the plates were centrifuged, and the supernatant was collected for cytokine measurements by ELISA. The pellet was lysed and suspended in 200 µL of phosphate-buffered saline. Next, 100 µL was transferred to BHI medium, and the colonies (CFU) were counted for 15 days. Data represent the means ± SE of the CFU from three donors, tested in triplicate. *p < 0.05, **p < 0.01, ***p < 0.001 by comparing the data indicated by the bars.

Since *P. brasiliensis* recognition was observed to be TLR-9-independent, we assessed the role of some CLRs in fungal recognition by pDCs. CLRs are a large family of calcium-dependent carbohydrate binding molecules expressed by macrophages, DCs and other leukocytes (29). The mannose receptor (MR), dectin-1, dectin-2, dectin-3, and mincle receptors are examples of CLRs involved in antifungal immunity, although their function and signaling mechanisms are still being clarified (30). Anti-dectin-1, dectin-2, dectin-3, and mincle antibodies were used to block these receptors during the interaction of pDCs with *P. brasiliensis* yeast cells. We also used piceatannol (10 µM), which blocks the activity of the Syk enzyme, involved in CLR signaling. The specific blockade of dectin 2 and Syk, but not dectin 1, reduced the fungicidal ability of pDCs. This indicated the involvement of dectin-2/Syk signaling during fungal recognition and activation of pDCs (Figure 5A). Importantly, the Syk inhibitor piceatannol did not compromise fungal growth even when a high concentration (100 µM) was used in the *P. brasiliensis* culture (Figure 5B). We also investigated the role of dectin-3 and mincle receptors during the recognition of *P. brasiliensis* yeast cells by pDCs, because both CLRs were not previously studied in innate immunity against *P. brasiliensis* infection. Interestingly, dectin-3 but not mincle blocked by specific antibodies prior to *P. brasiliensis* stimulation, improved the recovery of yeast cells from the pDC-*P. brasiliensis* cocultures (Figure 5C).

**Figure 6:**
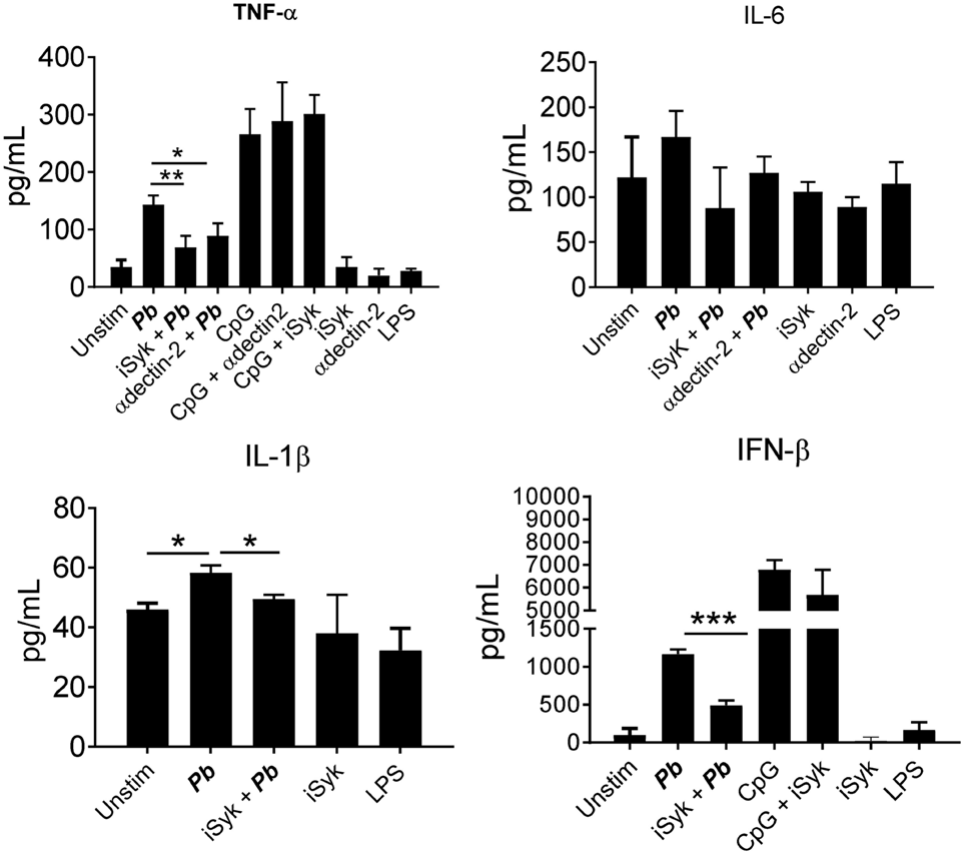
Dectin-2/Syk signaling controls the secretion of cytokines by *Paracoccidioides brasiliensis* stimulated plasmacytoid dendritic cells (pDCs). Peripheral blood mononuclear cells were separated into pDC-positive populations (pDCs^+^) using magnetic beads conjugated to anti-CD304 antibodies. The pDCs (1 × 10^5^/well) were challenged overnight with of *P. brasiliensis* yeast cells (2 × 10^3^). Some wells were kept only with the yeasts, CpG (100 ng/mL), or LPS (10 ng/mL). Some cultures were treated with anti-dectin-2 antibody (100 µg/mL) or with a Syk pathway inhibitor, piceatannol (10 µM), before challenging with *P. brasiliensis* yeast cells. After 18 h of culture, the plates were centrifuged and the supernatant was collected for cytokines measurements by ELISA. Data are represented by the means ± SE of the average cytokine concentrations from four donors, tested in duplicate. ***p < 0.001 and *p < 0.05 comparing the data indicated by the bars.

**Figure 7:**
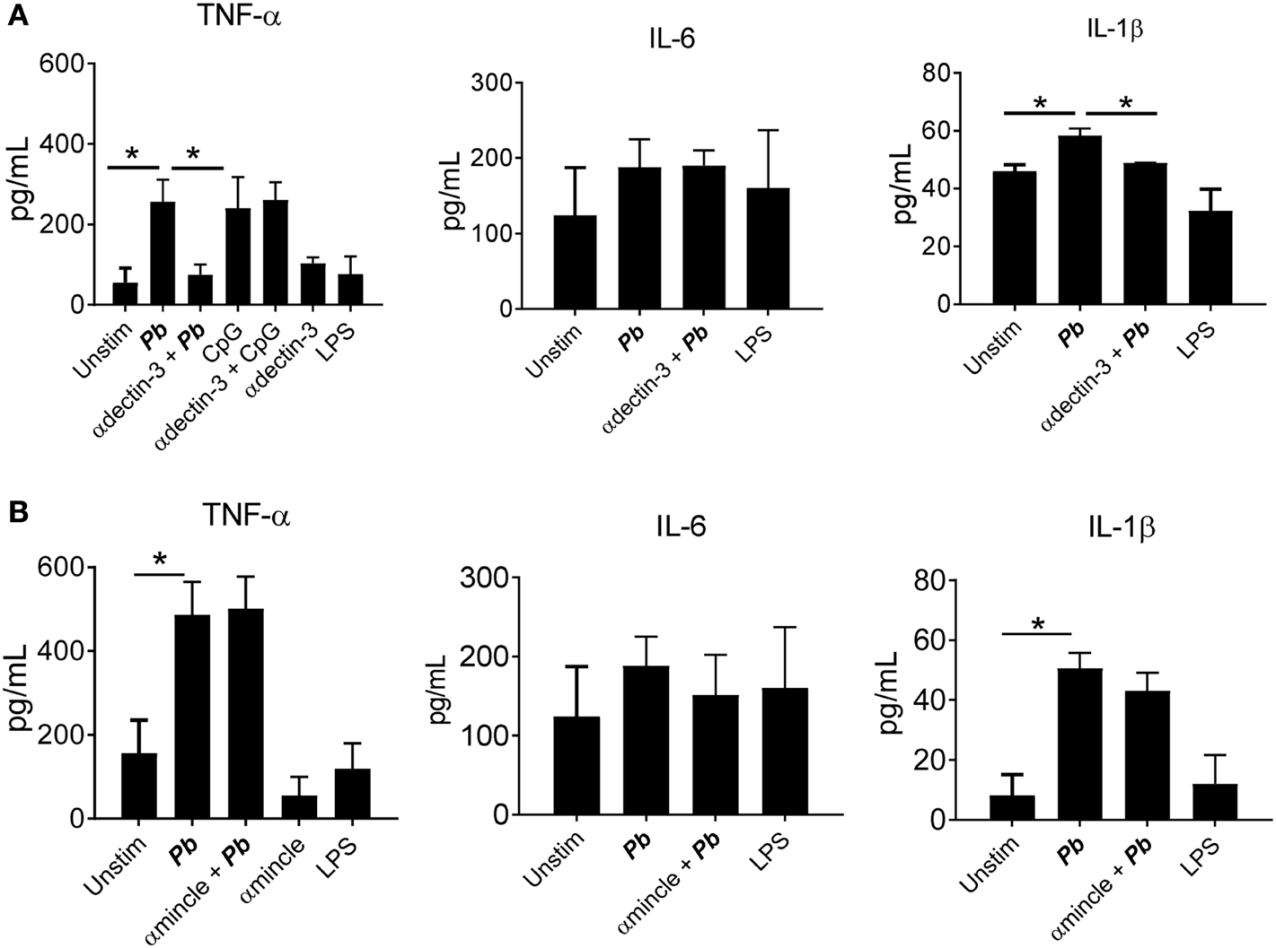
Dectin-3, but not mincle, controls cytokine secretion by *Paracoccidioides brasiliensis* stimulated plasmacytoid dendritic cells (pDCs). Peripheral blood mononuclear cells were separated into pDC-positive populations (pDCs^+^) using magnetic beads conjugated to anti-CD304 antibodies. The pDCs (1 × 10^5^/well) were challenged overnight with *P. brasiliensis* yeast cells (2 × 10^3^). Some wells were treated only with the yeasts or with LPS (10 ng/mL). Some cultures were treated with **(A)** anti-mincle (100 µg/mL) or **(B)** anti-dectin-3 antibody (100 µg/mL). After 18 h of culture, the plates were centrifuged, and the supernatant was collected for cytokine measurements by ELISA. Data are represented as the mean ± SE of the average of cytokine concentrations from four donors, tested in duplicate. ***p < 0.001 and *p < 0.05 comparing the data indicated by the bar.

Regarding cytokines released, an impaired production of TNF-α, IL-1β, and IFN-β was observed when dectin-2 or Syk were inhibited. No inhibition of IL-6 production was observed when the Syk pathway or dectin-2 were blocked (Figure 6). We also observed a reduction in the production of TNF-α and IL-1β following treatment with anti-dectin-3 antibody (Figure 7A). In line with the CFU results, treatment with the anti-mincle antibody did not influence cytokine production by *P. brasiliensis*-stimulated pDCs (Figure 7B). Again, treatment with both anti-dectin-3 and mincle antibodies did not alter IL-6 production. Importantly, in contrast with the absence of blocking activity on pDC, the anti-mincle antibody blocked mDC (Figure S2 in Supplementary Material).

**Figure 8:**
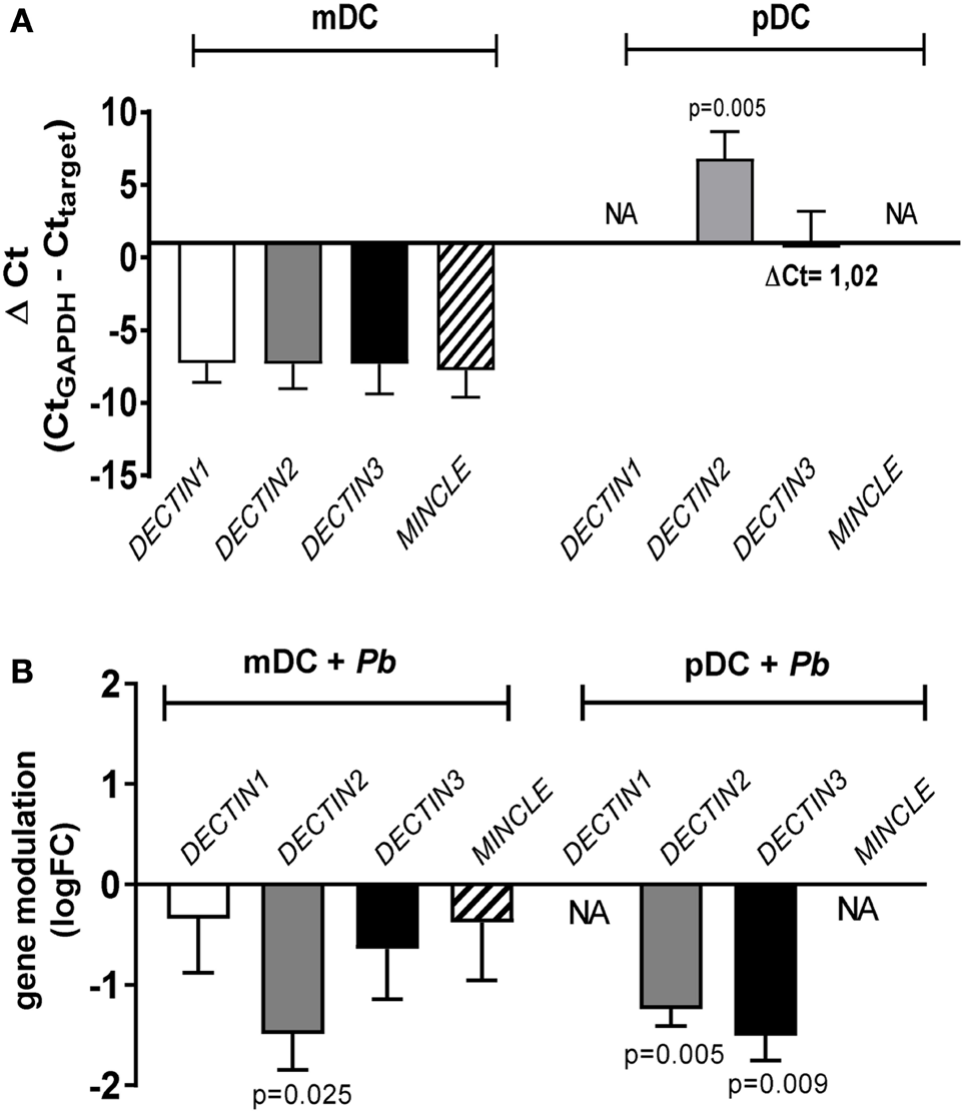
DECTIN1/2/3 and MINCLE gene expression in myeloid dendritic cell (mDC) and plasmacytoid dendritic cell (pDC) stimulated with *Paracoccidioides brasiliensis*. mDCs and pDCs from four healthy individuals were either left unstimulated or challenged overnight with *P. brasiliensis* yeast cells. Then RNA was isolated from each condition and used to evaluate the expression of DECTIN1/2/3 and MINCLE genes by qPCR. GAPDH was used as housekeeping gene for raw data normalization. **(A)** Basal genes expression in mDC and pDC was calculated as ΔCt (ΔCt = Ct_GAPDH_ − Ct_target_) as previously reported (31). Data are represented as mean (ΔCt) ± SE. Kruskal–Wallis test was applied to verify the significance of the difference between basal gene expression in mDC and pDC. When significant, the ΔCt of mDC versus pDC genes is indicated at the top of each bar (*p*). NA, not amplified. **(B)** The modulation of expression by *P. brasiliensis* was (20) calculated using the 2−ΔΔCt (fold-change, FC) method comparing stimulated and unstimulated cells (ΔΔCt = ΔCtPb − ΔCtUnstimulated). Data are represented as mean (logFC) ± SE. Student’s *t*-test was applied to verify the significance of the FC. When significant, the fold-change of stimulated versus unstimulated cells is indicated at the top of each bar (*p*).

When the expression of *DECTIN1, DECTIN2, DECTIN3* and *MINCLE* genes was investigated in mDCs and pDCs, we observed that while all genes were expressed in mDCs, only *DECTIN2* and *DECTIN3* were found on human pDCs, therefore explaining the previously mentioned lack of effect of blocking antibodies for dectin-1 and mincle in pDC activation. The basal expression of *DECTIN2* resulted significantly higher in pDC compared to mDC (*p* = 0.005) (Figure 8A). When DCs where stimulated by *P. brasiliensis* yeast cells, the expression of *DECTIN2* and *DECTIN3* resulted downregulated compared to unstimulated resting cells (FC < 1) (Figure 8B). Of note *P. brasiliensis* induced a statistically significant down-regulation of *DECTIN2* in mDCs (FC = 0.10 ± 0.08; logFC = −1.49 ± 0.36; *p* = 0.025), and of *DECTIN2* (FC = 0.07 ± 0.02; logFC = −1.24 ± 0.17; *p* = 0.005) and *DECTIN3* (FC = 0.05 ± 0.02; logFC = −1.51 ± 0.24; *p* = 0.009) in pDCs. These results highlight the importance of CLRs in *P. brasiliensis* recognition by human pDCs.

### pDC–Lymphocyte Interaction

**Figure 9:**
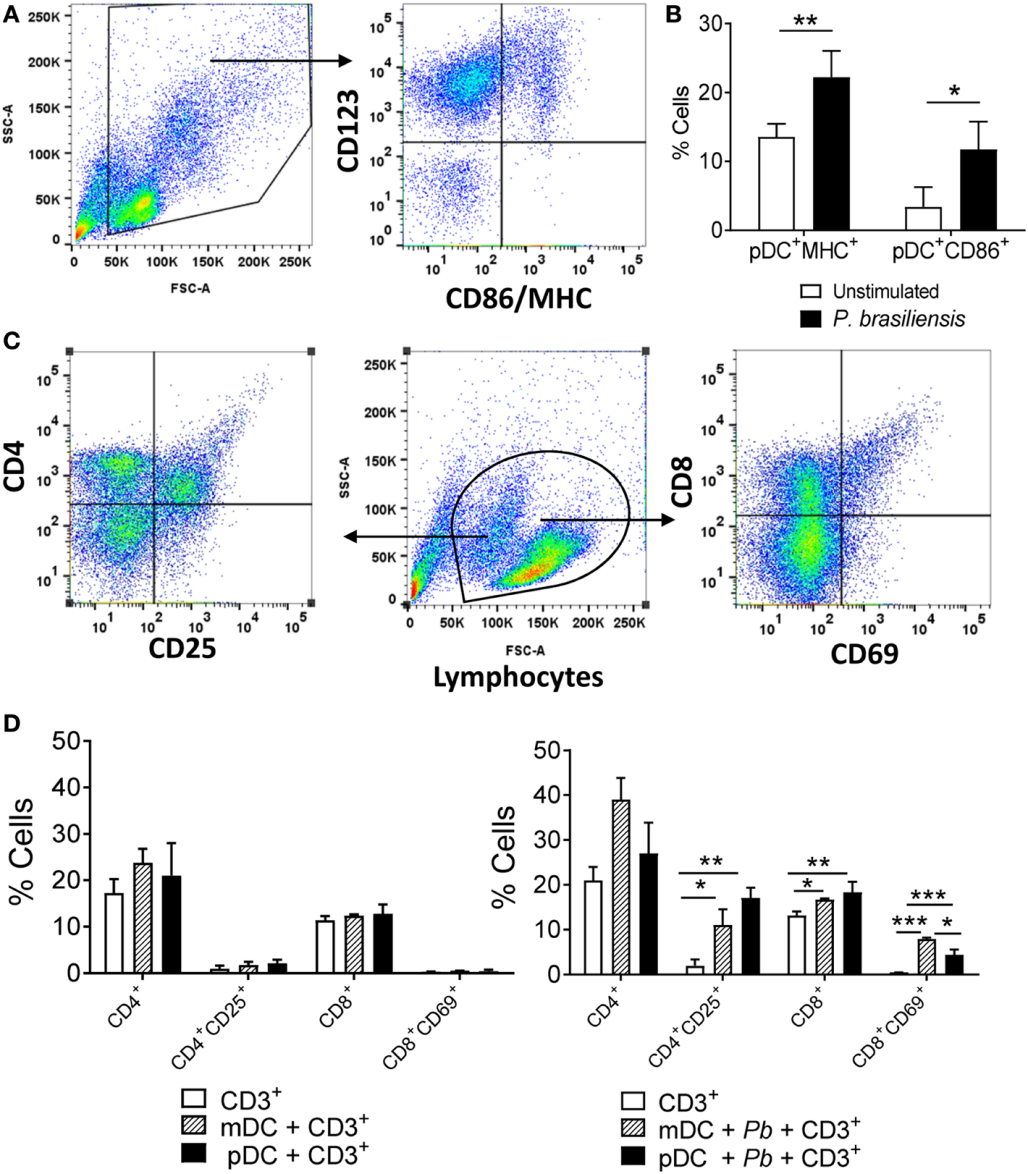
*Paracoccidioides brasiliensis* stimulation induces a mature phenotype in plasmacytoid dendritic cells (pDCs) that become able to promote the activation of CD4^+^ and CD8^+^ T lymphocytes. Peripheral blood mononuclear cells (PBMCs) were separated into pDC-positive (pDCs^+^) and myeloid dendritic cell (mDC)-positive (mDC^+^) population using magnetic beads conjugated to anti-CD304 and anti-CD1c antibodies, respectively. **(A,B)** For characterization of pDCs activation, the cells were labeled with appropriate titers of anti-CD123, MHC-class II, and CD86 antibodies and then 50,000 events were acquired in the flow cytometer and gated as indicated in the dot plots **(A)**. As the pDC^+^ population was stained with an specific pDC marker (anti-CD123), the characterization of cell maturation was performed by obtaining the pDCs from the PBMCs following one round of positive selection. Data are the means ± SE of the frequency of double-positive cells from three donors, tested in triplicate **(B). (C,D)** For coculture experiments, the pDCs and mDCs (1 × 10^5^/well) were cultured with or without *P. brasiliensis* yeast cells (1 × 10^4^) and after 2 h they were then placed in cocultured with lymphocytes (1 × 10^6^), which were previously obtained from PBMCs of the same donor and separated with anti-CD3 magnetic beads. After 5 days of coculture, the lymphocytes were analyzed by flow cytometry using anti-CD4, CD8, CD25, and anti-CD69 antibodies. The control wells contained only CD3^+^ cells. The lymphocyte population was gated by FSC/SSC analysis and the gated cells were then analyzed for expression of lymphocyte activation markers **(C)**. Data are the means ± SE of the frequency of single positive (CD4^+^/CD8^+^) double-positive cells (CD4^+^CD25^+^ and CD8^+^CD69^+^) from three donors, tested in triplicate in cultures with [**(D)**, right panel] or without *P. brasiliensis* yeast cells [**(D)**, left panel]. *p < 0.05, **p < 0.01, ***p < 0.001 by comparing the data indicated by the bars.

Additional studies demonstrated that *P. brasiliensis*-challenged pDCs showed an increased expression of membrane molecules typically found in activated APCs. *P. brasiliensis*-stimulated pDCs presented a higher expression of both MHC-class II and the costimulatory molecule CD86 when compared with unstimulated counterparts (Figures 9A,B). Since this result indicated that the pDCs transitioned to a mature status upon *P. brasiliensis* stimulation, we investigated the role of pDCs as APCs. In this regard, we explored the priming of antigen-specific CD4^+^ and CD8^+^ T lymphocytes *in vitro*. The pDCs^+^ and mDCs^+^ populations were each challenged with *P. brasiliensis* yeast cells and cocultured with CD3^+^ cells for 5 days. The mDC-CD3 coculture was used as a positive control because the priming ability of mDCs to specific T lymphocytes was previously described (15). We verified that both pDCs and mDCs recognized, processed, and presented *P. brasiliensis* antigens to the T lymphocytes. A higher frequency of activated CD4^+^ T lymphocytes (CD4^+^CD25^+^) was observed in both cultures maintained with mDCs and pDCs compared to the group of lymphocytes cultured without *P. brasiliensis* stimulation. A similar response was found for CD8^+^ T lymphocytes; however, the sensitized mDCs expanded a higher frequency of activated CD8^+^ T lymphocytes (CD8^+^CD69^+^) compared to the pDCs (Figures 9C,D, right panel). Importantly, the CD3^+^ cells did not respond to the DC stimulation in the absence of *P. brasiliensis* in the coculture (Figure 9D, left panel).

## Discussion

The involvement of pDCs has been prominently described in viral infections (5). However, recent studies have opened new roles of pDCs to other types of pathogens, indicating an important role for these cells in bacterial (32, 33) and fungal infections (8–12, 34). However, to our knowledge, no data are available regarding the role of human pDCs in innate and adaptive immunity against *P. brasiliensis*. Here, we show that human pDCs inhibit the growth of *P. brasiliensis* yeast cells. They also mature and produce cytokines upon stimulation with the yeasts. In addition, we have also demonstrated that dectin-2 and dectin-3 (*via* Syk signaling) are pattern receptors involved in *P. brasiliensis* sensing by human pDCs.

Studies with FITC labeled yeasts showed that about 15% of pDCs interact with yeast cells. As flow cytometric assays can not distinguish between adherent and ingested yeasts we can not guarantee that the yeasts were phagocytosed by the pDCs. However, our further experiments showed that the lymphocytes activation promoted by the pDCs was *P. brasiliensis*-specific indicating that pDCs can phagocytose, process and present fungal antigens to the lymphocytes. In addition, CpG, a TLR-9 agonist, has moderately increased the adhesion/engulfment of *P. brasiliensis* by pDCs. Although TLR-9 is not a phagocytic receptor, it is possible that the activation of pDCs *via* TLR-9 activates the cells and triggers mechanisms that promote the ability of pDCs to recognize the fungus. More importantly, we show in this study that human pDCs control fungal growth. Ramirez-Ortiz et al. (9) were the first authors to describe a non-redundant role for human pDCs during host defense against a human fungal pathogen. Human pDCs spread over *A. fumigatus* hyphae, inhibited their growth and released inflammatory cytokines (9). A recent report has also shown that mouse and human pDCs have cytotoxic activity against the opportunistic fungal pathogen *Cryptococcus neoformans*. The mechanism involves reactive oxygen species activity, which is distinct from that engaged by pDCs to control *A. fumigatus* infections (35).

The recognition of *P. brasiliensis* by human pDCs leads to TNF-α secretion. Although the pDCs were able to produce IL-6 in response to an infection (36), the levels of this cytokine did not increase following *P. brasiliensis* stimulation. The release of TNF-α by human pDCs is in agreement with the previous report regarding *A. fumigatus* hyphae stimulation (9, 10). In addition, in a mouse model of PCM, our group verified that *P. brasiliensis* infection induced BMDCs of resistant A/J mice to generate a population of pDCs that secreted inflammatory cytokines, including TNF-α (8). This was the first evidence that pDCs were involved in the host response to *P. brasiliensis*. We have also demonstrated that mouse pDCs release TNF-α and type I IFNs in response to *P. brasiliensis* yeast cells (34), a finding confirmed in the current study of human pDCs. Although the roles of type I IFN and pDCs are well known in DNA and RNA viral infections (36), their function in fungal infections is poorly defined. Romani et al. did not observed IFN-α secretion by pDCs stimulated by *A. fumigatus* resting conidia (37) but the tissue invasive hyphal morphotype was shown to stimulate human pDCs to release IFN-α (9, 10).

Our previous studies showed that dectin-1 and Syk signaling activate the NLRP3 inflammasome in *P. brasiliensis* stimulated macrophages (16). Furthermore, type I IFNs were shown to induce the expression of inflammasome components such as those of the NLRP3, retinoic acid-inducible gene 1, and AIM2 (38). This was in addition to the up-regulation of caspase-11, an enzyme required for the non-canonical activation of the NLRP3 inflammasome (39). These findings led us to investigate whether pDCs were able to activate the inflammasomes, activate caspase-1 and release IL-1β after *P. brasiliensis* stimulation. Our results showed that pDCs are capable of IL-1β release and caspase-1 activation, indicating that *P. brasiliensis* activates the pDCs inflammasome. This is a finding never described previously with regard to this DC subset during a host–microbe interaction.

Several PRRs, including dectin-1 (15, 17, 40), TLR-2 (13, 40), TLR-4 (14, 15, 40) and TLR-9 (41, 42) have been reported to recognize *P. brasiliensis* components. Human pDCs express several PPRs such as DC immunoreceptor, dectin-2, and siglec-H, but do not express TLR-2, TLR-4, and the CLRs dectin-1, MR, and DC-SIGN (43–46). In a previous study, we verified that dectin-2 participates in the interaction of pDC and *A*. *fumigatus*-hyphae and that this recognition leads to TNF-α and IFN-α release by the pDCs as well as improved antifungal resistance (10). In this study, we have also investigated the role of some PRRs in *P. brasiliensis* recognition by pDCs. Our studies show that TLR-9 does not play a role in the *P. brasiliensis*–pDC interaction. These results are similar to those found during the studies with *A. fumigatus* hyphae in which, the hyphae–pDC interaction was also TLR-9 independent (9). Although TLR-9 was reported as an important PRR in the control of murine PCM (41, 42), our data indicate that TLR-9 expressed by other innate immune cells was possibly responsible for this effect.

The cytoplasmic moieties of CLRs have an immunoreceptor tyrosine-based activation motif. These moieties engage the Syk tyrosine kinase, which transduces signals that activate the transcription factor NF-kB and downstream gene expression. This results in increased phagocytosis and cytokine production by innate immune cells (47). During *C. albicans*-hyphae recognition, dectin-3 forms heterodimers with dectin-2, compared with their respective homodimers, the dectin-3-dectin-2 heterodimers bind α-mannans more effectively, leading to potent inflammatory responses (48). Our results showed the involvement of dectin-2/dectin-3/Syk signaling during *P. brasiliensis* recognition by human pDCs.

As studies regarding the expression of CLRs on human pDC are scarce and sometimes conflicting (49), we have decided to investigate the gene expression for the dectin-1, dectin-2, dectin-3, and mincle receptors on human pDC upon *P. brasiliensis* stimulation. While all genes studied were expressed on human mDCs, only *DECTIN2* and *DECTIN3* were found on human pDCs. Although some studies have demonstrated the expression of dectin-1 on human pDCs (50) the expression of this receptor was not observed in our study. The absent expression of *DECTIN1* and *MINCLE* explained the lack of effect of blocking antibodies during pDC activation by *P. brasiliensis* yeast cells. Importantly, a limitation of our study is that we did not analyze the expression of these receptors on cell membrane of DCs after stimulation with the fungus. However, it is known that the endocytosis of plasma membrane-localized PRRs after a microbial encounter hinders their detection (51, 52). Notably, our findings are in agreement with a previous study of *A. fumigatus* in which fungal hyphae were recognized by human pDCs through dectin-2 rather than dectin-1 or TLR-7/9 (9, 10). Although yeasts of *P. brasiliensi*s and *A. fumigutus*-hyphae are composed of different PAMPs (53–56), the involvement of dectin-2 and dectin-3 receptors in triggering pDCs responses following fungal stimulation indicates that these receptors play an important role in the innate immune response to fungal infections. Importantly, dectin-3 has also been shown to participate in the inhibition of *C. neoformans* growth by human and mouse pDCs (35). These previous findings and those reported here highlight the importance of some CLRs (mainly dectin-2 and dectin-3) in the fungal recognition response by human pDCs. However, mincle, another receptor belonging to the dectin-2 family of CLRs (57) was not observed to participate in *P. brasiliensis* sensing by human pDCs even though it is involved in fungal recognition by conventional DCs (44).

Importantly, curdlan (an agonist of dectin-1) was previously shown to induce the production and secretion of mature IL-1β by activating the cytosolic NLRP3 inflammasome and this process requires the phosphorylation of Syk kinase (58, 59). However, as our study indicated that human pDCs do not express dectin-1, it is possible that other CLRs *via* Syk signaling can be used by *P. brasiliensis-*stimulated pDCs to activate the inflammasomes pathway.

Although the fungicidal activity of pDCs observed in this study occurred only at very high concentrations of pDCs relative to the fungus, our results of pDC–yeast interaction and cytokine production brought original clues to elucidate the biological function of human pDCs in the immune response against *P. brasiliensis*. In addition, using the pDC-lymphocyte coculture experiments, we could demonstrate that this cell population can trigger an antigen-specific adaptive immune response. Mittelbrunn et al. were the first to show that mature pDCs form canonical immune synapses that involve the relocation of the microtubule-organizing center, F-actin and protein kinase C to the contact site, in addition to the activation of early signaling molecules in T cells (60). In pulmonary PCM, our group previously demonstrated that mouse pDCs exert an important regulatory function during the host defense process by preferentially expanding regulatory T lymphocytes in a mechanism dependent on the enzyme indolamine 2,3-dioxygenase-1, which confers a tolerogenic profile to pDCs (34). To our knowledge, the current work is in agreement with previous data showing the presence of pDC in cutaneous lesions and in the blood of PCM patients (11, 12), and this is the first study describing the ability of human pDCs to trigger adaptive immune responses in a context of non-viral infectious disease. However, additional studies are necessary to elucidate the patterns of T cell immunity induced by fungi-sensitized pDCs.

In conclusion, our data show for the first time that *P. brasiliensis* activates human pDCs that inhibit fungal growth and secrete pro-inflammatory cytokines such as TNF-α and type I IFNs. Surprisingly, *P. brasiliensis-*stimulated pDCs activate caspase 1 and produce mature IL-1β, possibly *via* inflammasome activation. This phenomenon has not been previously described during pDC engagement by a microorganism. Importantly, dectin-2 and dectin-3 (acting *via* Syk signaling) are PRRs expressed on pDC and are involved in the pDC–*P. brasiliensis* interaction that results in pDCs activation. Therefore, our data support an important role for CLRs in *P. brasiliensis* recognition by pDCs, indicating that this DC subset actively participate in the mechanisms of innate and adaptive immunity against this primary fungal pathogen.

## Ethics Statement

All research involving human participants was approved by the Institute of Biomedical Science Institutional Ethics Committee. Written informed consent was obtained from all human participants and all clinical investigations were conducted according to the principles expressed in the Declaration of Helsinki.

## Author Contributions

Conceived and designed experiments: NP, ACN, AP, VC, and FL. Contributed with reagent: AP. Performed the experiments: NP, CF, DSL, BS, and FL. Analyzed the data: NP, BS, and FL. Wrote the article: NP, DSL, AP, ACN, VC, and FL.

## Conflict of Interest Statement

The authors declare that the research was conducted in the absence of any commercial or financial relationships that could be construed as a potential conflict of interest. The reviewer PF and handling Editor declared their shared affiliation.
