# Supplementary material for: The Syk-Coupled C-Type Lectin Receptors Dectin-2 and Dectin-3 Are Involved in Paracoccidioides brasiliensis Recognition by Human Plasmacytoid Dendritic Cells
Source: Front Immunol. 2018 Mar 20;9:464. doi: 10.3389/fimmu.2018.00464 (PMC5869931; doi:10.3389/fimmu.2018.00464)
Supplement: Supplementary file 1 [file Data_Sheet_1.PDF]

Supplementary Fig 1

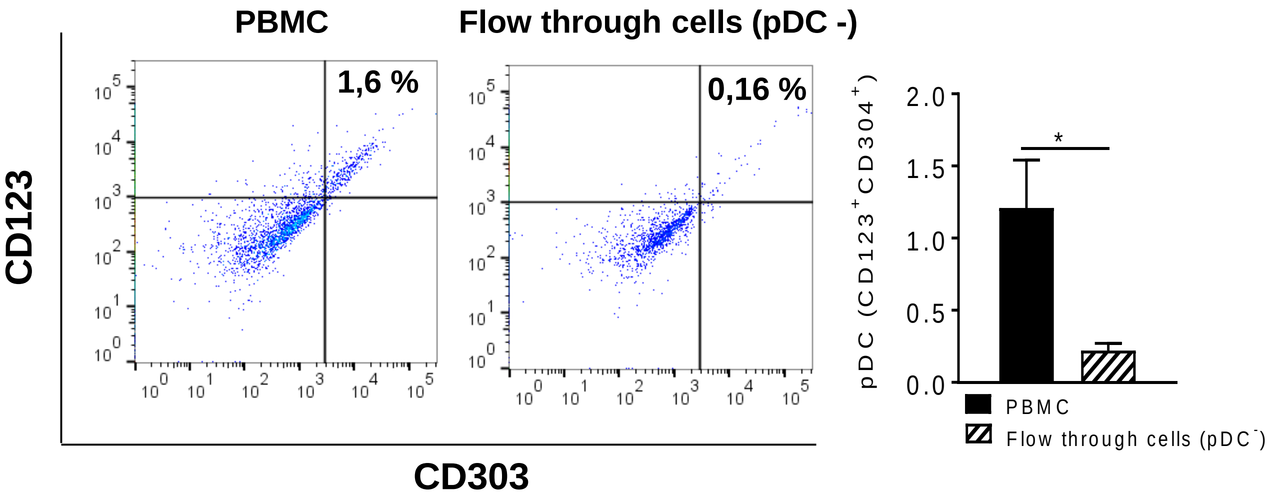

Supplementary Fig 1. PBMCs were separated into pDC-positive (pDCs+) and pDC-negative (pDC-) fractions using anti-CD304-coated magnetic beads. The PMBCs and flow through-cells ($1x10^5$/well) were also stained with anti-CD123 and anti-CD303 antibodies to evaluate the frequency of pDCs on PBMCs before and after the isolation process**.** Dot plots are representative results for an experiment from three independents experiments. Data represent means ± SE of the double-positive population from three donors, tested in triplicate. * p <0.05 by comparing the frequency of pDC between total PBMC and pDC-depleted PBMC.
