# Supplementary material for: The Syk-Coupled C-Type Lectin Receptors Dectin-2 and Dectin-3 Are Involved in Paracoccidioides brasiliensis Recognition by Human Plasmacytoid Dendritic Cells
Source: Front Immunol. 2018 Mar 20;9:464. doi: 10.3389/fimmu.2018.00464 (PMC5869931; doi:10.3389/fimmu.2018.00464)
Supplement: Supplementary file 2 [file Data_Sheet_2.PDF]

Supplementary Fig 2

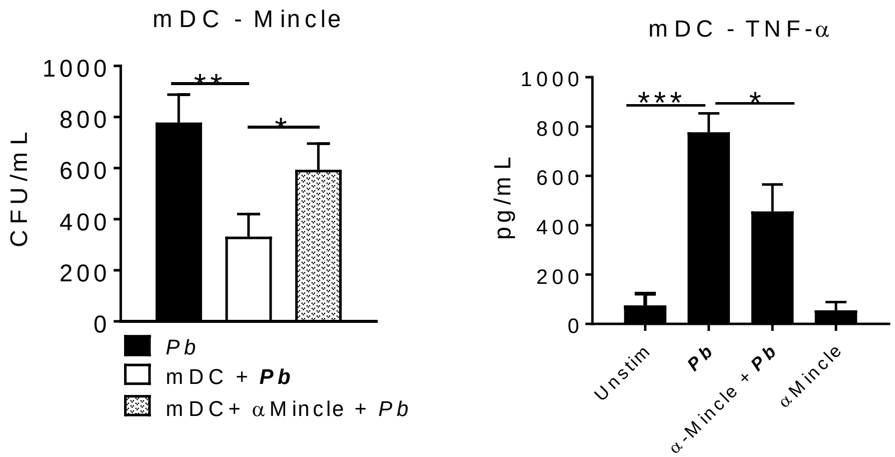

Supplementary Fig 2. Mincle activates the fungicidal mechanisms and controls the secretion of TNF-α by *P. brasiliensis*-stimulated mDCs. PBMCs were separated into mDC positive populations using magnetic beads conjugated to anti-CD1c antibody. The cells ($1x10^5$/well) were challenged overnight with *P. brasiliensis* ($2x10^3$) yeasts. Some cultures were treated with anti-mincle antibodies (100 µg/mL) before challenging with *P. brasiliensis* yeasts. After 18 hr of culture, the plates were centrifuged and the supernatant collected for cytokine measurements by ELISA. The pellet was lysed and suspended in 200 µL of PBS and 100 µL were transfered to BHI medium and the colonies (CFU) counted for 15 days. Data represent means ± SE of CFU from two donors, tested in triplicate. * *p* <0.05, ***p* <0.01, *** *p*<0.001 by comparing the data indicated by the bars.
